# Pathologic Collision of Inverted Papilloma with Esthesioneuroblastoma

**DOI:** 10.3389/fonc.2014.00044

**Published:** 2014-03-14

**Authors:** Sana D. Karam, Ann K. Jay, Cynthia Anyanwu, Matthew K. Steehler, Bruce Davidson, Pedro Debrito, K. William Harter

**Affiliations:** ^1^Department of Radiation Oncology, Georgetown University Hospital, Washington, DC, USA; ^2^Department of Radiology, Georgetown University Hospital, Washington, DC, USA; ^3^Department of Otolaryngology, Georgetown University Hospital, Washington, DC, USA; ^4^Department of Pathology, Georgetown University Hospital, Washington, DC, USA

**Keywords:** inverted papilloma, esthesioneuroblastoma, skull base, collision tumor, sinonasal malignancy, head and neck cancer

## Abstract

**Background:** Inverted papilloma (IP) of the nasal cavity is a benign tumor that represents 0.5–4% of all nasal tumors and have been known to rarely undergo malignant transformation to squamous carcinoma and even more rarely adenocarcinoma. Synchronous association with low-grade esthesioneuroblastoma (ENB) has been reported in only one case report where a small-sized lesion was treated with surgery alone. Here we report the first case of invasion of IP by high-grade ENB with nodal metastasis that was treated with combined modality therapy.

**Case Presentation:** A case of a 64-year-old African American gentleman presented to the otolaryngology with a 3-month history of recurrent epistaxis. Imaging revealed a large right nasal cavity mass extending into the right sphenoid sinus but without intracranial extension. Surgical pathology revealed high-grade ENB invading IP. An orbitofrontal craniotomy approach was used to achieve complete resection of the mass but with positive margins. Post-operative positron emission tomography/computed tomography showed nodal metastasis. The patient was then treated with adjuvant chemoradiation and remains without evidence of disease at 42 months post-treatment. We discuss the disease presentation, histopathologic features, and disease management with literature support.

**Conclusion:** In this very rare disease presentation where two extremely rare malignancies collide, we show that aggressive management with trimodality therapy of surgery, adjuvant radiation with stereotactic radiosurgical boost, and adjuvant chemotherapy gives excellent results. Given the natural history of the disease, however, long follow-up is needed to declare complete freedom from the disease.

## Introduction

Inverted papilloma (IP) of the nasal cavity is a benign but locally aggressive, benign nasal lesion remarkable for its tendency for local recurrence. These tumors represent 0.5–4% of all nasal tumors and are associated with sinonasal squamous cell carcinoma in approximately 5% of the cases and even more rarely adenocarcinoma (1). Esthesioneuroblastoma (ENB) is another uncommon malignancy of the head and neck, representing only 3–6% of all nasal cavity and sinonasal neoplasms (2). It is a tumor of neural crest origin that is believed to arise from the olfactory epithelium. Based on the low incidence of ENB and IP, the odds of these two unrelated tumors occurring in the same person in the same location are exceedingly rare. In the existing literature, synchronous association of IP with ENB has been reported in only one case report where a small-sized lesion was treated with surgery alone (3). In this paper, we present a case report of a collision of the high-grade ENB with IP in a patient treated with definitive surgical resection with positive margins and post-operative findings of nodal metastasis. He was treated with adjuvant chemoradiation followed by stereotactic radiosurgery boost to high-risk disease. We discuss disease presentation, pathological findings, and treatment strategy.

## Case Presentation

**Figure 1:**
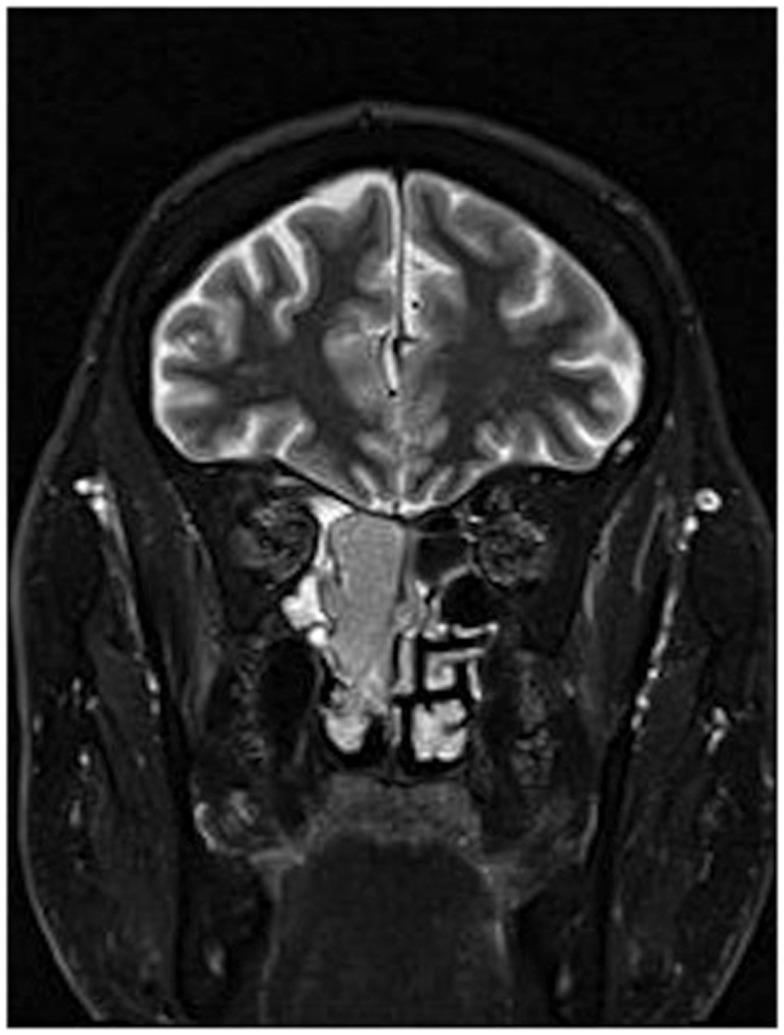
**MRI of esthesioneuroblastoma/inverted papilloma collision tumor**. Mass can be visualized in addition to inspissated secretions in the surrounding sinuses.

**Figure 2:**
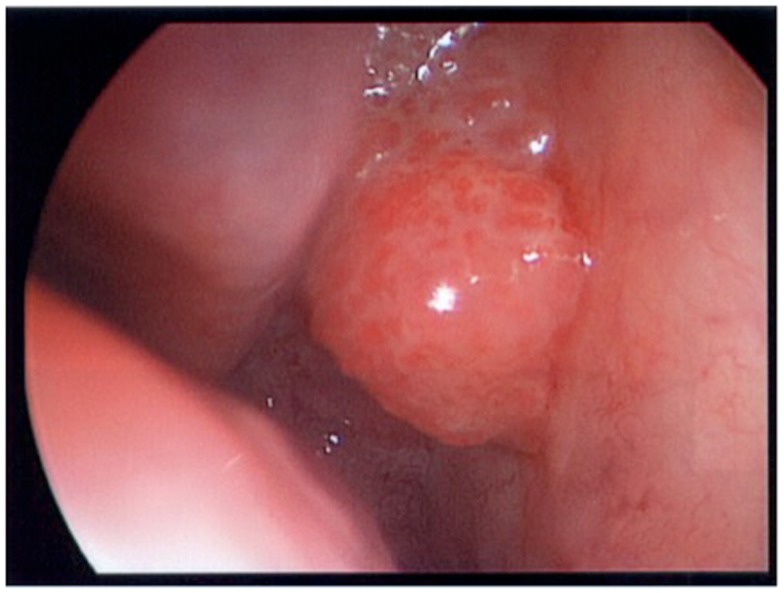
**Nasal endoscopy view of collision tumor**.

**Figure 3:**
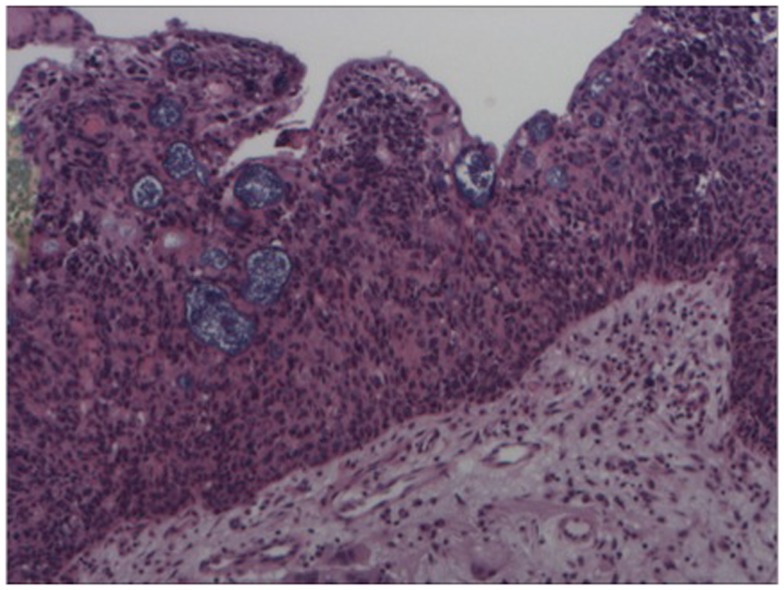
**Hematoxylin and eosin stain – high power of sinonasal inverted papilloma with infiltration of epithelium by atypical cells (esthesioneuroblastoma)**.

**Figure 4:**
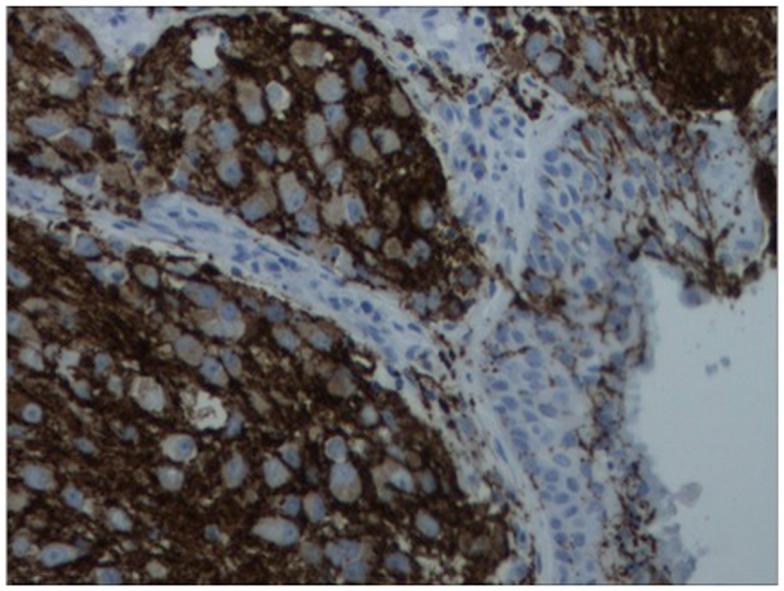
**Immunohistochemistry of lesion for synaptophysin (characteristically stains positive in esthesioneuroblastoma)**.

**Figure 5:**
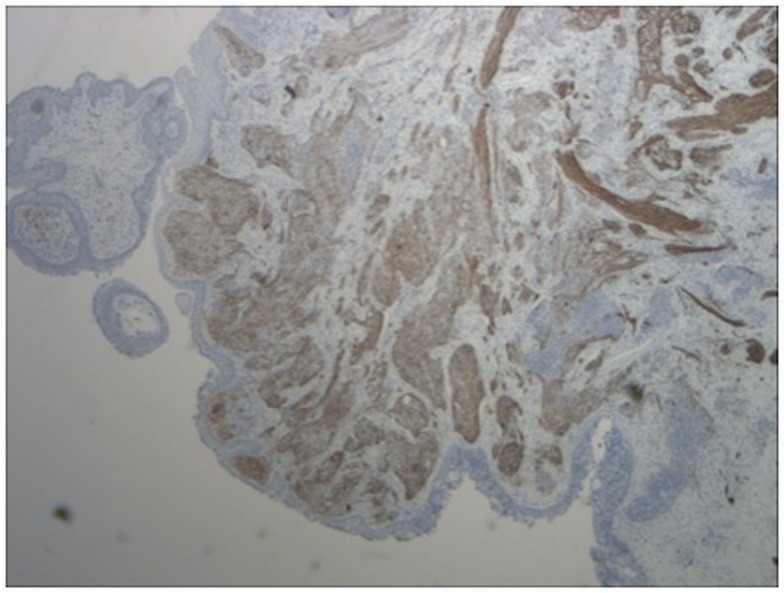
**Immunohistochemistry of lesion for neurofilament stain (characteristically stains positive in esthesioneuroblastoma)**.

A 64-year-old African American gentleman presented to otolaryngology head and neck surgery clinic with a 3-month history of recurrent epistaxis. On physical examination and nasal endoscopy, the patient had a right nasal cavity mass. Computed tomography (CT) revealed a right nasal cavity lesion extending into the ethmoid and sphenoid sinuses. The mass was locally destructive, with bony remodeling surrounding it. It measured 2.9 cm in the transverse dimension by 3.9 cm in the cranial–caudal dimension by 6.6 cm in the anterior–posterior dimension. On MRI, the mass obstructed the right sphenoid sinus with inspissated secretions surrounding the mass (Figure 1). The mass extended up to the cribriform plate but without evidence of intracranial extension or orbital involvement. Nasal endoscopy (Figure 2) with debulking and biopsy revealed a high-grade malignant neoplasm adjacent to a sinonasal papilloma with focal inverted growth pattern (Figure 3). The tissue was negative for Epstein–Barr virus nuclear antigen-1 (EBNA-1) by polymerase chain reaction (PCR) and Epstein–Barr virus encoded RNA (EBRER) by immunohistochemistry. Further immunohistochemical analysis showed the neoplastic cells to be focally positive for pan-keratin, neuron-specific enolase (NSE), synaptophysin (Figure 4), epithelial membrane antigen (EMA), and vimentin. Additionally, rare immunoreactivity was noted in the subepithelial tumor cells for chromogranin, neurofilament (Figure 5), and GFAP. The specimen tested negative for leukocyte common antigen (LCA), latent membrane protein (LMP), S-100, CD20, CD3, desmin, and smooth muscle actin. The Ki-67 proliferation index was reported at approximately 70%. The immunohistochemical profile is consistent with a diagnosis of a collision tumor of high-grade ENB with extensive involvement of overlying schneiderian papilloma.

### Treatment

Surgical resection was performed via anterior and subfrontal craniotomy. Sphenoid sinus disease was described to attach anteriorly through the sphenoid ostium but without direct evidence of sphenoid mucosal disease. Complete surgical resection was reported. Pathological examination, however, showed positive margins on the superior border of the resection. Given this margin positivity and high-grade pathological features, the decision was made to pursue adjuvant concomitant chemoradiation, which was initiated following his surgical recover at 9 weeks post-operatively. Positron emission tomography (PET)/CT scan conducted for radiation treatment planning revealed increased metabolic uptake in the bilateral levels I and II neck nodes consistent with nodal metastasis. His final stage was T2N1M0.

**Figure 6:**
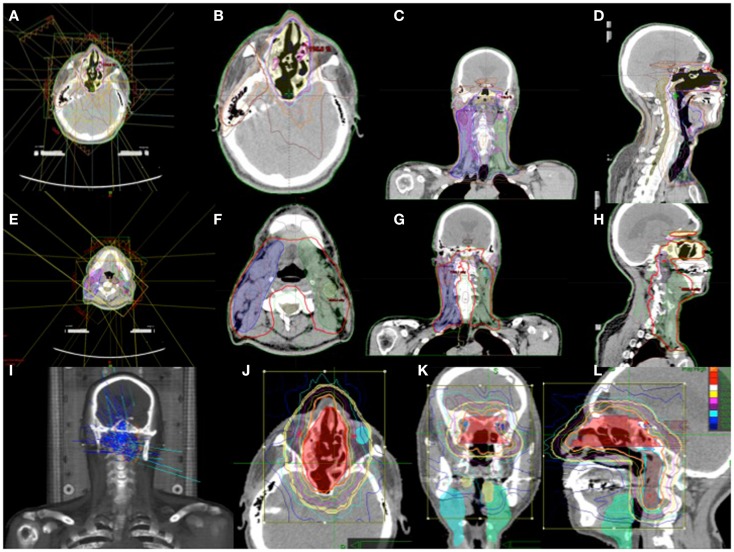
**(A–H) IMRT treatment plan with contoured target volume in axial, coronal, and sagittal view for the nasopharyngeal volume (A–D) and the neck volumes (E–H)**. **(I–L)** Cyberknife treatment plan with the target volume in axial, coronal, and sagittal views.

The determination of target volumes was based on pre-operative and post-operative CT scans, 1 mm MRI images with VIBE sequence, and thin sliced PET/CT complemented by clinical and endoscopic information. The target volume included the regions of high-risk and regions of lower-risk for microscopic tumor spread. This consisted of the surgical bed with margins, bilateral comprehensive lymph node volume levels 1–5 including all PET positive disease, and the bilateral retropharyngeal lymph node volumes. Using 6 mV photon energies, the plan was designed with intensity modulated radiation therapy (IMRT) to improve conformality and reduce dosage to critical structures. A prescription was written for 28 fractions in 180 cGy each for a total dose of 5040 cGy. Due to temporary machine malfunction during the treatment, dose adjustment was made for 29, 180 cGy fractions for a total dose of 5220 cGy. This was completed over a period of 6 weeks. Using stereotactic radiosurgery with Cyberknife (Accuray Inc.), a reduced field was designed to the surgical bed and bilateral retropharyngeal lymph node volumes in five fractions of 250 cGy each. The bilateral PET positive levels I and II neck nodes were then boosted with 6 mV photons with IMRT in five fractions of 250 cGy. The total cumulative dose was 6470 cGy. Figure 6 shows a composite of his radiation treatment plan.

Concomitant chemotherapy included cisplatin (33 mg/m^2^) and etoposide (100 mg/m^2^) for three cycles during weeks 1, 4, and 7 of the radiation treatment. He suffered the expected toxicity from the chemoradiation with intractable nausea, vomiting, grade III mucositis, and dysphagia. He was treated symptomatically but required two hospitalizations for failure to thrive and symptom management. His pain was managed with patient-controlled analgesia pump and for nutritional supplementation, total parenteral nutrition was initiated toward the end of his treatment. On his 3-month visit following the end of his radiation treatment, he had fully recovered from the rigors of the treatment. At his 42 months follow-up visit, his clinical examination and all of his radiographic imaging studies including MRI and PET/CT were without evidence of disease.

## Discussion

Inverted papillomas have been historically considered true neoplasms due to their locally aggressive nature and tendency for local recurrence (4); new concepts in the pathogenesis of IPs suggest that they may instead be an end stage of a chronic inflammatory condition rather than a true neoplasm (5). It is estimated that 11% of recurrent IPs undergo malignant transformation (6). Human papillomavirus (HPV) serotypes 6, 11, 16, and 18 have been detected in these lesions. Low risk serotypes 6 and 11 are associated with benign papillomas; while serotypes 16 and 18 are associated with higher risk of malignant transformation and recurrence (6).

Collision of IP with ENB is an extremely rare event. Synchronous association has only been reported once in the literature where IP collided with low-grade ENB in a case of less aggressive disease presentation (3). ENBs are typically considered low-grade tumors that respond well to treatment and immunohistochemical confirmation has been recommended in cases of pathologic findings of high-grade ENBs to avoid initial misdiagnosis and to treat aggressively (7). IHC demonstrates that greater than 90% of ENB cells are neuron-specific enolase positive. Approximately 80% of cases are positive for S-100, staining cell nests, and synaptophysin (8). Cytokeratin AE1/AE3 and EMA are typically negative with ENB (9). Unlike sinonasal undifferentiated carcinoma, ENB demonstrates lesser pleomorphism, may contain smaller nucleoli, and is EMA-negative and S-100 positive (10).

The diagnosis of a high-grade ENB has been shown to have a significant impact on survival (7, 11, 12). In a retrospective review by Dias and colleagues (13), the 5-year disease-specific survival (DSS) for patients with low-grade tumors was 64%, whereas for patients with high-grade tumors it was 43%. The importance of a patient’s tumor histopathologic findings, as they relate to prognosis, vary among reports. Morita and colleagues (14) examined the pathologic findings of 49 patients with ENB and noted the pathologic grade correlated with prognosis. Levine and colleagues (15) found no valuable pathologic or molecular indicators to predict aggressive clinical behavior in their series of patients. Recently, however, comparative mutational genomic analysis of samples taken from a patient with ENB at the time of initial presentation and following recurrent metastatic disease showed new acquired mutations in KDR, MYC, SIN3B, and NLRC4 genes with disease progression (16). Kim et al. (17) examined 17 ENB specimens for staining with bcl-2, p53, MIC-2, and N-myc. Of note, 70% of specimens were positive for bcl-2. All specimens were negative for N-myc. MIC-2 and p53 staining was noted in only one specimen. The results suggested a potential survival advantage and improved response to chemotherapy with bcl-2 positivity in patients with ENB, yet this finding was not statistically significant.

Lymph node metastasis is also an important prognostic factor as the 5-year survival rate is 64% for node-negative disease compared to 29% for those with lymph node metastases (2, 15, 18). Most series report that less than 15% of individuals present with regional nodal metastasis at the time of initial evaluation (2, 15, 18). Zefereo et al. (19) noted that 22% their patients were stage N1 at diagnosis. These series, however, are based on clinical staging where most patients had not received PET scanning. Wu and colleagues (20) showed ENB was PET positive in seven of nine patients (77.7%) with a maximal standard uptake value (SUV max) of 6.37–4.22 in primary tumors. Tracer uptake did not correlate with tumor size. PET/CT detected regional metastases in two (cervical and parapharyngeal) patients and distant metastases in four (lung, liver, and bone). PET/CT altered the clinical staging in three of the nine patients. The use of pretreatment PET/CT has also been advocated by other authors (21).

The gold standard for treatment of ENB is a combination of surgery and radiotherapy, with or without chemotherapy. Tumor stage, histopathologic grade, and use of adjuvant radiation were found to be important determinants of disease-free survival in a meta-analysis examining 26 studies (from 1990 to 2000) with 390 patients (2). Adjuvant radiation dosing of 55 Gy or greater was an additional variable found to be of prognostic value in a large retrospective analysis that examined the outcomes of 77 patients with non-metastatic ENB from the Rare Cancer Network (22). Adjuvant radiation has been shown to improve local control after surgery with a decrease in local recurrence rates from 71 to 17%, as well as decrease the risk of regional recurrence (23). The timing of radiation, before or after surgery, does not seem to impact the benefit it offers to local disease control (24). Stereotactic radiation dose escalation has been shown to yield excellent survival outcomes without optical toxicity. Nichols and colleagues (25) reported on the use of adjuvant proton beam radiation in 10 patients with ENB with predominantly Kadish stage C disease. With a median follow-up of 52.8 months, 5-year DFS and OS rates were 90 and 85.7%, respectively. In a more aggressive combination of proton therapy with neoadjuvant chemotherapy, Fitzek and colleagues also reported actuarial 5-year survival of 74% (26).

Chemotherapy has been generally accepted for patients with ENB with high-grade, recurrent, or unresectable disease (27). The combination of cisplatin and etoposide represents a popular regimen that has been successfully used in the neoadjuvant and adjuvant setting. A review from the Mayo Clinic, involving 12 patients with Kadish stage C high-grade ENB, examined the use of adjuvant cisplatin and etoposide after complete surgical resection. Six patients received adjuvant chemotherapy with radiation and six received only post-operative radiation. The addition of adjuvant chemotherapy improved median time to relapse from 10.5 to 35 months, yet did not significantly affect OS (28).

## Conclusion

In this paper, we reported a very rare and unique pathologic presentation of a collision of IP and ENB. The pathological and immunohistochemical features were diagnostic of high-grade ENB, which drove the clinical management of the disease. The patient received complete surgical resection with adjuvant chemoradiation for positive microscopic margins and high-grade pathological features. Radiation dose escalation to areas of high-risk disease was achieved with a combination of IMRT and SBRT. At 42 months post-treatment, the patient remains without evidence of disease. However, long term follow-up (>10 years) remains the ultimate test in determining the success of this therapy given the extended time to local and regional recurrence of ENB (2).

## Conflict of Interest Statement

The authors declare that the research was conducted in the absence of any commercial or financial relationships that could be construed as a potential conflict of interest.
